# mTOR pathway and Ca^2+^ stores mobilization in aged smooth muscle cells

**DOI:** 10.18632/aging.100555

**Published:** 2013-05-08

**Authors:** Francisco E Martín-Cano, Cristina Camello-Almaraz, David Hernandez, Maria J Pozo, Pedro J Camello

**Affiliations:** Department of Physiology, Faculty of Nursing and Faculty of Veterinary Sciences, University of Extremadura, 10003 Cáceres, Spain

**Keywords:** mTOR, Ca^2+^ signal, FKBP12, colon, smooth muscle

## Abstract

Aging is considered to be driven by the so called senescence pathways, especially the mTOR route, although there is almost no information on its activity in aged tissues. Aging also induces Ca^2+^ signal alterations, but information regarding the mechanisms for these changes is almost inexistent. We investigated the possible involvement of the mTOR pathway in the age-dependent changes on Ca^2+^ stores mobilization in colonic smooth muscle cells of young (4 month old) and aged (24 month old) guinea pigs. mTORC1 activity was enhanced in aged smooth muscle, as revealed by phosphorylation of mTOR and its direct substrates S6K1 and 4E-BP1. Mobilization of intracellular Ca^2+^ stores through IP_3_R or RyR channels was impaired in aged cells, and it was facilitated by mTOR and by FKBP12, as indicated by the inhibitory effects of KU0063794 (a direct mTOR inhibitor), rapamycin (a FKBP12-mediated mTOR inhibitor) and FK506 (an FKBP12 binding immunosuppressant). Aging suppressed the facilitation of the Ca^2+^ mobilization by FKBP12 but not by mTOR, without changing the total expression of FKBP12 protein. In conclusion, or study shows that in smooth muscle aging enhances the constitutive activity of mTORC1 pathway and impairs Ca^2+^ stores mobilization by suppression of the FKBP12-induced facilitation of Ca^2+^ release.

## INTRODUCTION

Aging can be viewed as a quasi-programmed process driven by changes in signaling pathways, among which the mTOR pathway stands out as a key factor [1; 2]. This kinase forms part of a energy-sensing signaling pathway and can assemble two types of heteromeres, mTORC1 and mTORC2, the former involved in aging based on the lifespan-extending effects of the mTOR inhibitor rapamycin (reviewed in [3]). mTORC1 is activated by nutrients, insulin and the PI_3_K/Akt pathway, and can be inhibited by nutrients deprivation, AMPK activation [4] and FKBP12 [5; 6], the target for the immunosuppressant rapamycin. mTOR has been proposed to convert arrest to senescence (geroconversion) [7; 8]. In spite of this, there is very little information regarding the mTOR pathway status in aged tissues [9; 10].

One of the functional consequences of aging is the alteration of different aspects of calcium signals, which play a key role in multiple cellular functions, from contraction or secretion to gene regulation and cell fate and proliferation. Therefore, these changes are the basis for important alterations linked to aging [11-15]. Calcium signals consist of cytosolic Ca^2+^ concentration ([Ca^2+^]_i_) increases due either to activation of Ca^2+^ entry from extracellular space (through Ca^2+^ channels activated by voltage, Ca^2+^ stores depletion or receptors), or to Ca^2+^ release from intracellular stores. Mobilization of intracellular stores is mediated by IP_3_R and RyR channels, activated respectively by the intracellular messengers IP_3_ and cADPribose. The subsequent [Ca^2+^]_i_ decrease to resting levels is operated by active extrusion to extracellular medium or reuptake into intracellular organelles.

Most of available evidence on the effects of aging on [Ca^2+^]_i_ has focused on Ca^2+^ clearing mechanisms [15-17] and influx of extracellular Ca^2+^ [15; 18; 19], but it is rather limited and controversial for Ca^2+^ release. In neurons, the most studied model for age-related changes in Ca^2+^ signals, there is evidence that aging enhances Ca^2+^ release from intracellular stores through RyR and IP_3_R calcium channels [13], similar to reports in cardiomyocytes [20]. In aged smooth muscle cells both inhibition [21] and enhancement [22; 23] have been reported.

Regarding the mechanisms underlying the effects of age on [Ca^2+^]_i_ signals, knowledge is rather superficial, most of the studies addressing only a description of the signal alterations. Some authors have proposed that mitochondrial modifications or redox imbalance are the origin for Ca^2+^ signal disfunction [14; 24]. Interactions between some mTOR pathway elements and Ca^2+^ release imply that the mTOR pathway could be involved in the age-related [Ca^2+^]_i_ modifications: Ca^2+^ release from intracellular stores can be modulated by mTOR kinase [25-28], and its inhibitor FKBP12 is a known modulator of Ca^2+^ release channels [29]. More explicit, a recent study hypothesizes that in aging neurons down-regulation of FKBP12 activates mTOR and enhances RyR mediated Ca^2+^ release [5], explaining the Ca^2+^ signal phenotype typical of aged neurons [12].

The aim of this study was to investigate the involvement of mTOR and FKBP12 in the age-induced changes in Ca^2+^ release in smooth muscle cells. Our data indicate that aging reduces the intracellular Ca^2+^ release, an effect accompanied by loss of a facilitatory effect of FKBP12 on Ca^2+^ mobilization which was independent on the expression of this protein. mTOR facilitated Ca^2+^ release both in young and aged cells and showed enhanced activity in aged cells.

## RESULTS

### Effect of aging on mTOR pathway activation

**Figure 1:**
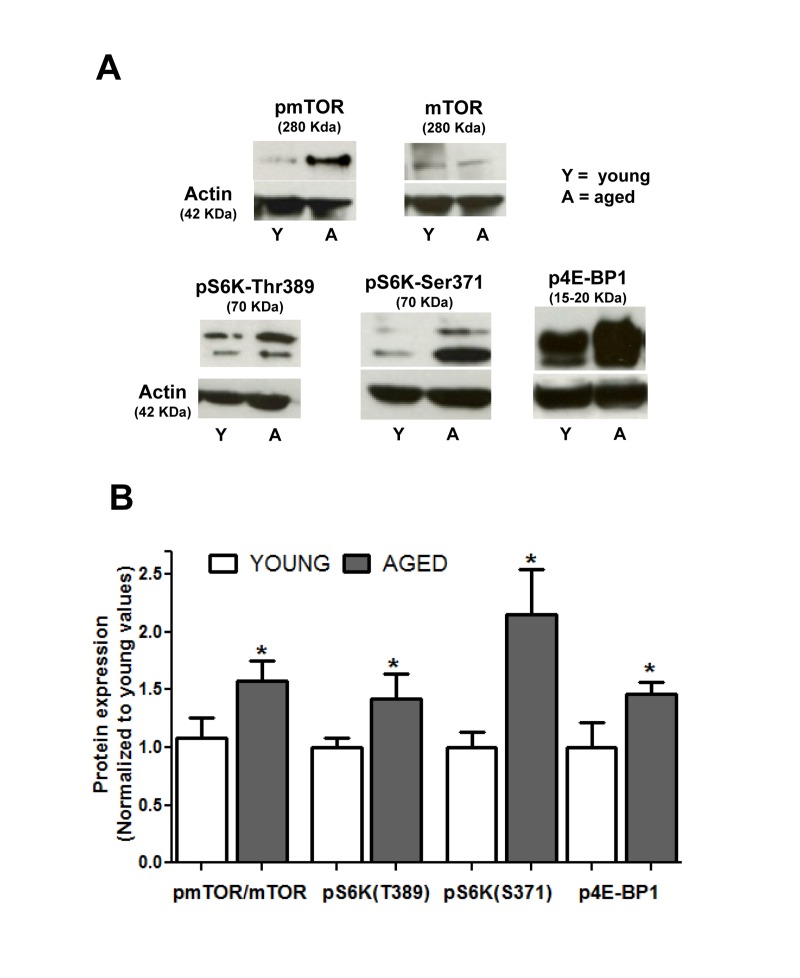
Aging enhances the constitutive activity of the mTOR pathway in colon smooth muscle (**A**) Representative immunoblotts showing expression of mTOR and phosphorylated (active) forms of mTOR, p70S6K1 (at Thr389 and Ser371) and 4E-BP1 in non stimulated muscular layer of colon. Figures in parenthesis are the molecular weight of each band as judged from the molecular weight marker run in each assay. (**B**) Histogram depicting phosphorylation of mTOR and its substrates in young and aged colon. Values are average ± sem of arbitrary units normalized to mean average values of young colon. * p< 0.05, n = 6.

Since changes in the mTOR route have been proposed to be associated to the aging process [30], we investigated the constitutive activation of this pathway in the non stimulated smooth muscle layer of guinea pig colon. Activation of mTORC1 can be reliably determined by phosphorylation of Ser 2448 [31]. Figure 1 shows that Ser2448 phosphorylation was significantly enhanced in the aged colon muscle layer compared to the young colon (*p* < 0.05). To confirm that mTOR pathway was enhanced in aged muscle, we determined the level of phosphorylation of two direct substrates of mTOR, p70S6K1 and 4E-BP1, which are respectively activated and inhibited upon phosphorylation by mTORC1 kinase activity. We found that aged muscle showed higher levels of the phosphorylated forms of both 4E-BP1 and p70S6K1, the latter including phosphorylation of two different residues specific for mTOR kinase activity [32] (Fig. 1).

### Modification of Ca^2+^ signals in aged smooth muscle cells

**Figure 2:**
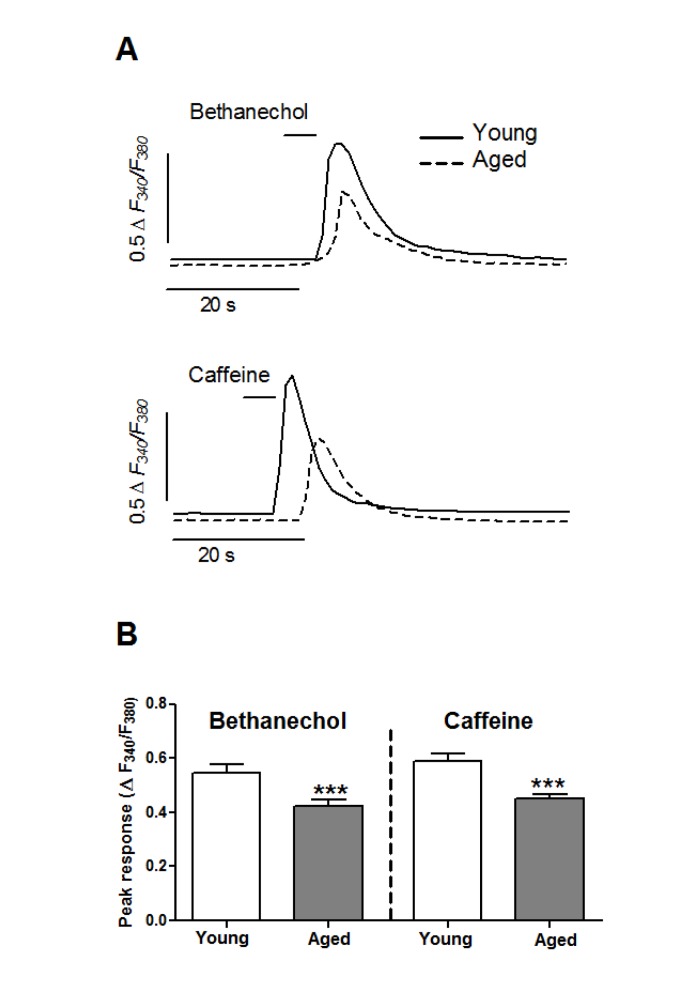
Aging inhibits the mobilization of Ca^2+^ stores in colonic smooth muscle cells (**A**) Isolated cells were challenged with a short pulse of bethanechol (0.1 mM) or caffeine (10 mM) to release Ca^2+^ from intracellular pools through IP_3_R and RyR channels, respectively. Traces are representative of average responses in young and aged cells. (**B**) Average ± sem response (ΔF_340_/F_380_) from young (8 animals, 117 and 165 cells) and aged (6 animals, 188 and 286 cells) guinea pigs.

To study the effects of aging in the mobilization of Ca^2+^ from intracellular stores we challenged colon smooth muscle cells with bethanechol, known to release Ca^2+^ through IP_3_R in this cell type [33; 34], or with caffeine, which releases Ca^2+^ through RyR [35]. [Ca^2+^]_i_ signals induced by mobilization of intracellular Ca^2+^ pools in smooth muscle cells are characterized by an initial [Ca^2+^]_i_ peak followed by gradual decline towards pre-stimulus levels, as shown in Figure 2. Bethanechol caused a transient [Ca^2+^]_i_ increase which was smaller in aged (0.418 ± 0.024 ΔF_340_/F_380_, n =1 88) than in young cells (0.546 ±0 .028 ΔF_340_/F_380_, n = 117, p <0.005). Compared to bethanechol, the effect of caffeine in young cells was also transient but larger in amplitude (0.588 ± 0.027 ΔF_340_/F_380_, n = 165, p < 0.05), and showed a similar decrease in aged cells (0.450 ± 0.018 ΔF_340_/F_380_, n = 286, p <0.005).

**Figure 3:**
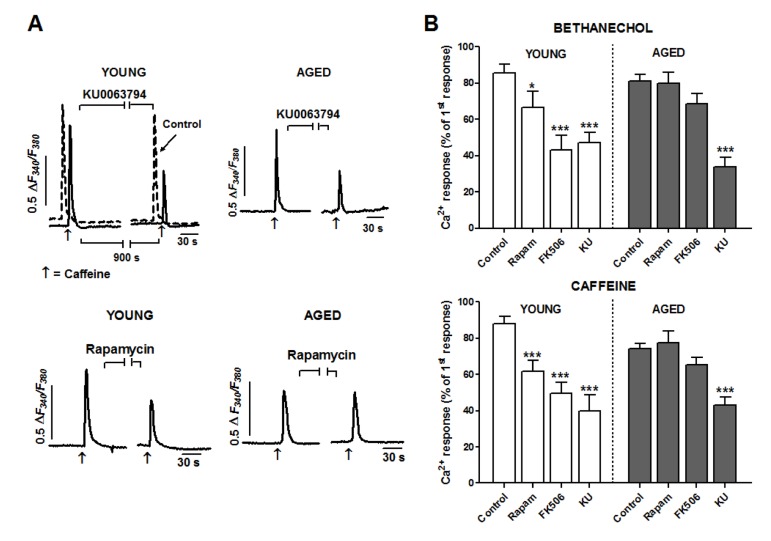
Aging reduces facilitation of Ca^2+^ release by FKBP12 but not by mTOR kinase in smooth muscle cells (**A**) Cells were stimulated with two pulses of caffeine (10 μM separated by a 15 min interval to apply inhibitors (indicated by horizontal lines) or normal medium (control trace). For the sake of comparison, the control trace in top left panel is shifted to the left. Traces are representative of the effects of KU0063794 (5 μM) or rapamycin (5 μM) on young (left column) or aged cells (right column). (**B**) Histogram summarizing the effects of the three inhibitors (KU: KU0063794, 5 μM; rapamycin 5 μM; FK506 10 μM) on the bethanechol (0.1 mM) and caffeine (10 mM) evoked Ca2+ responses in young and aged cells. Two-ways ANOVA showed significant effect for treatment (bethanechol: F = 22.4, p < 0.005; caffeine: F = 21.9, p < 0.005), which was modified by age (bethanechol: F = 4.0, p < 0.01; caffeine: F = 5.0, p < 0.01). Asterisks denote significance for planned comparisons between groups of interest. * p<0.05, ** p< 0.01, *** p< 0.005. n= 17-130 cells from 6 (aged) or 8 (young) animals.

To assess a possible role of mTOR in Ca^2+^ pools mobilization, we used a double pulse protocol: either bethanechol or caffeine was applied twice separated by a 15 minutes interval to allow for recovery of the response and acute application of inhibitors (see Fig. 3). In control experiments the amplitude of the second stimulus was close to 90 % respect to the first one in young cells (bethanechol: 85.60 ± 4.82 % respect to the first pulse, n = 54; caffeine: 88.10 ± 4.16 %, n = 74), and close to 80% in aged cells (bethanechol: 81.13 ± 3.59 %, n = 89; caffeine: 74.24 ± 2.87 %, n= 130) (Fig. 3).

To inhibit mTOR we used two different types of compounds: rapamycin, a frequently used mTOR inhibitor which binds to FKBP12 protein to inhibit mTOR kinase, and KU0063794, a second generation inhibitor acting directly at the kinase domain of mTOR. When applied to cells from young guinea pigs, KU0063794 (5 μM) significantly decreased (p < 0.005) the responses to bethanechol and to caffeine (by around 50% each, see Fig. 3). This inhibition was also present in aged cells, indicating that mTOR kinase activity has a facilitating role on IP_3_R- and RyR-mediated Ca^2+^ release and that this facilitation is not affected by aging. In the case of rapamycin (5 μM), its inhibitory effect in young cells was qualitatively similar to that of KU0063794 though smaller in amplitude (around 23 and 30% for bethanechol and caffeine, respectively; Fig. 3). On the contrary, in aged cells the inhibition was strongly attenuated or even suppressed, suggesting that the mechanisms of action of rapamycin on the Ca^2+^ mobilization are altered in aged cells.

To explore the differential influence of age on KU0063794 and rapamycin effects on Ca^2+^ mobilization, we treated colon muscle cells with the compound FK506, which binds FKBP12 without inhibiting mTOR activity. In young cells FK506 (10 μM) reduced clearly (p < 0.005) the responses to bethanechol and caffeine by approximately 50 and 60% respectively (Fig. 3). Similar to rapamycin, FK506 failed to inhibit significantly the responses in aged cells. Therefore, our data strongly suggest that aging alters the FKBP12-mediated facilitation of calcium signal, but not the facilitation induced by mTOR.

**Figure 4:**
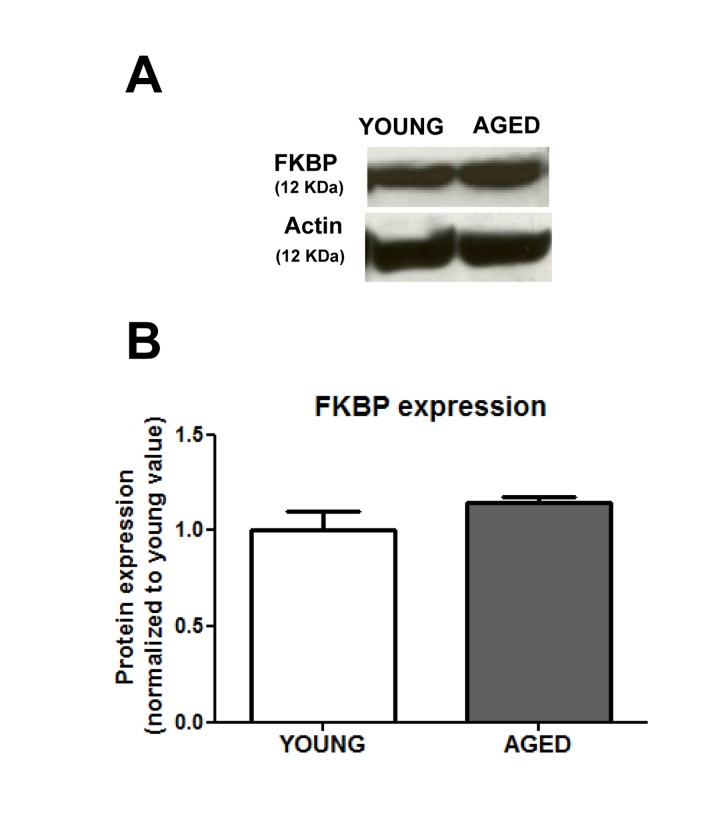
Aging does not change significantly the expression of FKBP12 protein (**A**) Representative immunoblotts and (**B**) average ± sem values (arbitrary units) (n=6) of the FKBP12 expression in muscular layer of colon from young and aged guinea pigs.

In view of our data, and given that a recent report shows an age-related decrease in FKBP12 expression [5], we explored a possible down-regulation of FKBP12 expression in the colonic smooth muscle layer of aged guinea pigs. Figure 4 shows, however, that in the aged colon there is a slight and non-significant (p < 0.09) increase in FKBP12 rather than a decrease in the expression of this protein.

## DISCUSSION

The present study shows that in colonic smooth muscle aging enhances the activity of the mTOR pathway and inhibits the mobilization of Ca^2+^ stores, an effect likely associated to the alteration of the facilitating effect of FKBP12 protein on Ca^2+^ mobilization.

Aging is considered a process regulated by longevity pathways among other factors. The mTOR route is an energy-sensing system whose inhibition with rapamycin prolongs lifespan in a number of models, resembling the effect of caloric restriction [3], hitherto the most successful intervention to extend lifespan. We describe here that in resting conditions aged smooth muscle cells show a significant increase in the expression and phosphorylation of mTOR, and in the phosphorylation of its targets p70S6K1 and 4B-EP1. To our knowledge, the only precedent of age-induced mTOR constitutive activity are hippocampal neurons in mice [9] and an enhancement of pS6 phosphorylation in male mice heart compared to females [10]. In human heart no effect of age has been reported [36]. Our findings are in keeping with a previous report showing increased mTOR pathway responses in senescent cultured cells [30]. Data from gene expression studies are conflicting. Rat hippocampus shows enhanced mTOR gene expression ([37], their supplementary data), but recent array studies on the expression of genes linked to the mTOR pathway in blood samples from human cohorts suggest that aging down-regulates this route [38], although this report found no decrease in mTOR gene expression for and Protor1, a protein forming mTOR complexes, was actually up-regulated. Another study in a cohort of long-lived nonagenarians [39] reported that increased longevity was associated to slightly reduced expression of mTOR gene, but the pattern of the gene expression for the mTOR complex proteins was unclear: for mTORC1 complex, protein Raptor was impaired but PRAS40 was enhanced, and for mTORC2 complexes only Protor2 was enhanced. Whether these discrepancies are due to methodological or to tissue-specific differences (see [9]) is unclear and need further investigation. In any case, our findings support the theory that aging is a quasi-programmed process driven by signaling routes [1]: enhanced mTOR levels would drive and accompany aging, so that the more long-lived individuals display lower levels [10, 38]. As inhibition of mTOR had the same influence in aged and young cells, it is unlikely, however, that our finding of an age-mediated decrease of Ca^2+^ release is due to the activation of mTOR pathway.

Given that Ca^2+^ signals are a key regulator in multiple cellular functions, age-related changes in this parameter are relevant for the aging process [15]. For example, age-related changes in Ca^2+^ influx [18; 19; 40] could reduce Ca^2+^ content of the stores, a condition known to alter several cellular functions [41], and can change gene expression [42; 43]. Although in striated muscle the effects of aging on Ca^2+^ signals seem to be relatively settled (enhanced Ca^2+^ sparks and impaired Ca^2+^ release upon stimulation; reviewed in [44]), reports in smooth muscle are very limited and somewhat conflicting. Initial contractility studies proposed enhanced release from intracellular stores in colon and arterial strips [22; 23] but not in gallbladder [45]. Results from [Ca^2+^]_i_ determination in isolated cells showed that aging decreases intracellular Ca^2+^ release in arterial and detrusor muscle cells [21; 46] but not in gallbladder cells [18]. We report here that in colonic smooth muscle cells aging impairs IP_3_R-and RyR-mediated Ca^2+^ release, suggesting that age influences this signaling system in a tissue-specific way.

At the moment there is very little information regarding the mechanisms causing age-related changes in Ca^2+^ signals. Because its facilitator role in Ca^2+^ release through IP_3_R and RyR channels [25; 27-29], modifications of mTOR and FKBP12 function are plausible candidates to mediate the age-induced Ca^2+^ signal changes. While there is no information regarding the mechanism of action of mTOR on Ca^2+^ release, the complex effects of FKBP12 are due to direct interaction with the channels and/or to inhibition of calcineurin, a known regulator of Ca^2+^ release. In fact, we describe here that both IP_3_ and RyR signals are impaired by inhibitors targeted to mTOR kinase (KU0063794), to FKBP12 (FK506) or to both (rapamycin, that binds FKBP12 to a regulatory domain of mTOR kinase). Our results agree with a previous work in colon myocytes for IP_3_-mediated signals [28], but are opposite for RyR-mediated signal [35]. The discrepancy could be due to differences in the age of the animals (we use 5 months old, more mature than other reports [35]) or to the experimental design (we use single pulses of stimulation instead of choosing cells responding to repetitive stimulation).

The finding that aging does reduce only the effect of the FKBP12-targeted inhibitors rapamycyin and FK506, indicates for the first time that aging could impair Ca^2+^ signals by alteration of the facilitation of Ca^2+^ release by FKBP12. On the contrary, the enhanced activity of mTOR and its facilitating effect on the Ca^2+^ signal in aged cells rules out an involvement of mTOR in the impairment of the Ca^2+^ mobilization. Although a recent report in hippocampus [37] describes that age-related Ca^2+^ deregulation is due to down-regulation of FKBP12, which operates there as an inhibitor of RyR-mediated Ca^2+^ release [5], we found that FKBP12 expression was not decreased in aged cells. Assuming that FKBP12 interaction to mTOR was normal in aged cells one would expect an inhibition of the signal comparable to that of KU0063794, which was not the case. It his therefore likely that aging simply alters the functional association of FKBP12 with Ca^2+^ release channels and even with mTOR activity. The differences in the effects of experimental manipulations of the mTOR pathway could be due to cell-specific changes in the regulation of aging. For example, in aged hypothalamus only some neuronal populations show enhanced mTOR activity in aged rodents [9]. The exact link between changes in FKBP12 expression and functional facilitation of Ca^2+^ release, out of the scope of the present study, deserves further investigation and could be related to the constitutive activation of mTOR pathway in aged cells.

In conclusion, this study shows that in smooth muscle the mTOR kinase activity is increased by aging, but although mTOR facilitates Ca^2+^ mobilization, aged cells present lower responses to agonists as the result of the loss of FKBP12-induced facilitation of Ca^2+^ release.

## METHODS

### Animals and cell isolation

Female guinea pigs (Dunkin-Hartley), housed in light (12 h light-dark cycle) and temperature (20°C) controlled conditions and with *ad libitum* access to water and food, were divided into two groups according to age: young adults (5 months old, average weight 1048.3 ± 82.7 g) and aged (28 month old, average weight 912.9 ± 22.6 g). The experiments were performed according to European guidelines for animal research and approved by the Animal Ethics Committees of the University of Extremadura.

Approximately 20 mg of the circular and longitudinal smooth muscle layer of the colon was cut into small pieces and incubated for 35 min at 37°C in enzyme solution (ES, for composition see *Solutions and drugs*) supplemented with 1 mg/mL BSA, 1 mg/mL papain and 1 mg/mL dithioerythritol. The tissue was then transferred to fresh ES containing 1 mg/mL BSA, 1 mg/mL collagenase and 100 μm CaCl_2_ and incubated for 10 min at 37°C. After washing with cold ES, single smooth muscle cells were mechanically isolated using a fire-polished pipette. Cell suspensions were kept in ES at 4°C until use, generally within 6 h. Cell viability (90%), as assessed by trypan blue staining was the same in all groups of animals.

### Cell loading and [Ca2+]i determination

[Ca^2+^]_i_ was determined by epifluorescence microscopy (Eclipse TE2000-S; Nikon, Melville, NY, USA) at room temperature using the ratiometric Ca^2+^ indicator fura-2. Isolated cells were loaded with 4 μM fura 2-AM at room temperature for 15 min. After loading, cells were perfused with Na^+^-HEPES solution in the absence or presence of the experimental agents. Cells were illuminated with a monochromator (Optoscan; Cairn Research, Faversham, UK) at 340-380 nm 1 Hz cycles, and the emitted fluorescence was captured with a digital camera (ORCAII-ERG; Hamamatsu Spain, Barcelona, Spain) and recorded using dedicated software (Metafluor; Universal Imaging, Molecular Devices, Downingtown, PA, USA). After background subtraction, fluorescence ratio (F340/F380) was calculated pixel by pixel and used to estimate the changes in [Ca^2+^]_i_. A calibration of the ratio for [Ca^2+^]_i_ was not performed in view of the many uncertainties related to the binding properties of fura 2 with Ca^2+^ inside of smooth muscle cells.

### Analysis of protein expression and phosphorylation by Western blot

Small pieces (~2 mg of dry weight) were quickly frozen, pulverized in liquid nitrogen, extracted in lysis buffer (for composition see *Solutions and drugs*) and then sonicated for 5 s. Lysates were centrifuged at 10000 g for 15 min at 4°C to remove nuclei and intact cells and the protein concentration was measured. Protein extracts (30 μg) were heat-denatured at 95°C for 5 min with DTT, electrophoresed on 7.5% and 15% polyacrylamide-SDS gels and then transferred to nitrocellulose membranes. Membranes were blocked for 1 h at room temperature using 10% bovine serum albumin (BSA) and incubated overnight at 4°C with affinity-purified polyclonal antibodies against mTOR, phospho-mTOR (Ser2448), phospho-4E-BP1, phospho-p70 S6 kinase (Ser371 and Thr389) (Cat#9862, Cell Signaling, Boston, MA, USA) and FKBP12 (#PA1-026A, Thermo Scientific, MA, USA).

A mouse anti-actin antibody (A2066, Sigma-Aldrich) was used to control for protein loading and to normalize expression of proteins of interest. After washing, the membranes were incubated for 1 h at room temperature with anti-mouse (1:10000; Amersham Biosciences, Bucks, UK) or anti-rabbit (1:7000; Santa Cruz Biotechnology) IgG-horseradish peroxidase conjugated secondary antibodies.

Bands were visualized using the supersignal west pico chemiluminescent substrate (Pierce, Rockford, IL, USA), quantified using the software gel-pro analyzer (4.0, Media Cybernetics, Bethesda, MD, USA) and normalized to α-tubulin content. For comparison purposes between young and aged samples, all the α-tubulin-corrected values were normalized to the average of young samples of the same assay.

In mTOR phosphorylation assay, two similar gels were run and one membrane was incubated with the antibody against the total protein and the other with an antiphospho-protein of interest.

### Solutions and drugs

Na^+^-HEPES solution (in mM): 10 HEPES, 140 NaCl, 4.7 KCl, 2 CaCl_2_, 2 MgCl_2_ and 10 D-glucose (pH 7.3). The Ca^2+^ -free Na^+^-HEPES solution included EGTA (1 mm) instead of CaCl_2_. ES (in mM): 10 HEPES, 55 NaCl, 5.6 KCl, 80 sodium glutamate, 2 MgCl_2_ and 10 D-glucose (pH 7.3). Lysis buffer (in mM): Tris-HCl 40, NaCl 400, 0.2% SDS and 10% glycerol supplemented with protease and phosphatase inhibitors (4-(2-aminoethyl) benzenesulfonyl fluoride, E-64, bestatin, leupeptin, aprotinin and sodium vanadate).

Drugs and chemicals were obtained from the following sources: bethanechol, caffeine and phosphatase inhbitors from Sigma-Aldrich (Madrid, Spain); fura 2-AM from Molecular Probes (Life Technologies, Madrid, Spain), rapamycin and KU-63794 from Calbiochem (VWR, Madrid, Spain), FK-506 from Cayman (VWR, Madrid, Spain), collagenase from Fluka (Madrid, Spain) and papain from Worthington Biochemical (Lakewood, NJ,USA).

### Quantification and statistics

Results are expressed as means ± standard error of the mean (sem) of n cells or blots. [Ca^2+^]_i_ responses are expressed as increases in the ratio of fura-2 fluorescence (ΔF_340_/F_380_). To compare two groups, paired *t* test was used to assess the effect of treatment. The effects of age and experimental treatments were tested using a two-way analysis of variance, followed by planned comparisons between selected groups. Differences were considered significant at p < 0.05.
